# Diverse perspectives on respiratory chemoreceptor interactions: Resuscitating an expired debate

**DOI:** 10.1113/EP091689

**Published:** 2025-03-31

**Authors:** Nasimi A. Guluzade, Daniel A. Keir, Richard J. A. Wilson, Trevor A. Day

**Affiliations:** ^1^ School of Kinesiology Western University London Ontario Canada; ^2^ Hotchkiss Brain Institute and Alberta Children's Hospital Research Institute University of Calgary Calgary Alberta Canada; ^3^ Department of Physiology and Pharmacology, Cumming School of Medicine University of Calgary Calgary Alberta Canada; ^4^ Department of Biology, Faculty of Science and Technology Mount Royal University Calgary Alberta Canada

**Keywords:** chemoreceptor interactions, respiratory chemoreflexes

## 1 INTRODUCTION

Connections link a sequence of three related research papers. The central article which links the other two papers has been published in Experimental Physiology. In a Connections article, an author (or authors) of the central article outlines its principal novel findings, tracing how they were influenced by the first article and how the central article has contributed to the developments made in the third article. The author(s) may also speculate on the direction of future research in the field. Connections articles aim to set the research in a wide context.

## 2 RESPIRATORY CHEMORECEPTOR INTERACTIONS

The chemosensory control of breathing maintains acute blood gas homeostasis through inputs from central (brainstem) and peripheral (carotid body) chemoreceptors. These chemoreceptor inputs, in concert with a variety of other afferent inputs (e.g., descending cortical control, lung stretch, airway receptors, joint/muscle receptors, baroreceptors), interact within brainstem respiratory centres to regulate ventilatory output. Synergistic combinations of inputs may change the responsiveness and/or activation threshold of brainstem respiratory centres to chemoreceptor inputs, affecting how ventilation responds to acute and chronic blood gas challenges.

These interactions between respiratory chemoreceptor inputs have been described in the literature as additive or multiplicative, with multiplicative interactions being hypo‐additive (i.e., antagonistic) or hyper‐additive (i.e., synergistic). Many contradictory reports have been published on how central and peripheral chemoreceptor inputs interact to control breathing involving a variety of model systems and methodologies. Model systems in published reports include rodents, dogs, goats and humans, and range from reduced preparations to intact animals and humans. Protocols range from artificial perfusion of organs to steady‐state, transient or rebreathing methods, eliciting inspired gas challenges. Briefly, steady‐state methods are used to hold chemoreceptor compartments at constant levels, transient methods utilize dynamic gas perturbations (e.g., a few breaths of low O_2_ or high CO_2_), and rebreathing methods utilize incremental increases in chemostimuli (e.g., CO_2_) with the metabolic rate into a circuit over a range of chemostimuli. These perturbations can be used to characterize the resulting respiratory responses.

Here we connect a series of experimental reports of respiratory chemoreceptor interaction, illustrating previous work in animals to more recent work in humans, with the aim of highlighting the use of creative protocols in humans from our group to attempt to reconcile disparate findings, and plot a way forward.

## 3 PREVIOUS EVIDENCE OF HYPER‐ADDITIVE INTERACTIONS IN DOGS USING TRANSIENT PERTURBATIONS OF THE CENTRAL CHEMOREFLEX

Using a unilaterally carotid body‐denervated, extracorporeally perfused carotid body, but otherwise systemically intact canine preparation, Blain et al. (2010) provide evidence of a hyper‐additive central–peripheral chemoreceptor interaction in the awake dog. Following normoxic/normocapnic carotid body perfusion (i.e., baseline), they silenced (hyperoxic–hypocapnic perfusion) or activated (hypoxic–normocapnic perfusion) the carotid body, while superimposing step‐wise increments in the fraction of inspired CO_2_, presumably stimulating the central chemoreceptors. They demonstrate a hyper‐additive interaction, whereby specific carotid body inhibition blunts the central chemoreflex to increases in CO_2_, whereas specific carotid body activation augments the central chemoreflex. These results were confirmed in a subsequent study, whereby a similar hyper‐additive interaction was demonstrated, except the isolated carotid body was perfused with normoxic hypocapnic, normocapnic or hypercapnic perfusate (Smith et al., 2015).

Interestingly, the pattern of chemo‐stimulation induced by this experimental design is counter to what would be encountered in vivo, where it is far more likely for steady‐state central CO_2_ to be superimposed with dynamic carotid body activation or inactivation (e.g., breath hold, sigh). These differences in stimulation pattern may affect the resulting pattern of responses, but also make it difficult to reconcile with other models in reduced animal models (e.g., Day & Wilson, 2009) or those in humans.

**Connected Articles**

- Smith, C. A., Blain, G. M., Henderson, K. S., & Dempsey, J. A. (2015). Peripheral chemoreceptors determine the respiratory sensitivity of central chemoreceptors to CO_2_: role of carotid body CO_2_. *The Journal of Physiology*, *593*(18), 4225–4243.
- Milloy, K. M., White, M. G., Chicilo, J. O. C., Cummings, K. J., Pfoh, J. R., & Day, T. A. (2022). Assessing central and peripheral respiratory chemoreceptor interaction in humans. *Experimental Physiology*, *107*(9), 1081–1093.
- Guluzade, N. A., Huggard, J. D., Duffin, J., & Keir, D. A. (2023). A test of the interaction between central and peripheral respiratory chemoreflexes in humans. *The Journal of Physiology*, *601*(20), 4591–4609.

## 4 EVIDENCE OF SIMPLE ADDITION OR HYPO‐ADDITION USING TRANSIENT TESTS OF THE PERIPHERAL CHEMOREFLEX IN HUMANS

Triggered by the disparate findings between reduced animal models from our group and others, and a desire to extend previous work using transient tests of the peripheral chemoreflex in humans, we utilized the transient 100% N_2_ hypoxic ventilatory responses (HVR) test in a background of moderate inspired hypercapnia, to assess the putative interaction between chemoreceptors in humans (Milloy et al., 2022). We found that three consecutive breaths of 100% N_2_ (i.e., transient hypoxia) elicited a similar magnitude HVR, regardless of steady‐state inspired CO_2_. Of note, the tidal volume response to transient hypoxia in a background of 4% inspired CO_2_ was lower than in either a background of ambient air (i.e., 0% inspired CO_2_) or a background of 2% inspired CO_2_, suggesting the tidal volume responses were hypo‐additive (Milloy et al., 2022). Thus, in humans with steady‐state brainstem conditions, the interaction appears to be simple addition in ventilation, due in part to a hypo‐additive interaction in tidal volume. In this way, the ‘addition’ and ‘hypo‐addition’ perspectives are congruent, as simple addition in minute ventilation must mathematically contain hypo‐addition in at least one of the underlying respiratory variables (rate and/or volume).

Not tested in these experiments was the peripherally mediated HVR characterized against a background of brainstem hypocapnic/alkalotic conditions (e.g., following sustained hyperventilation), conditions that best demonstrate strong hypo‐additive interaction in an artificially perused rodent model (Day & Wilson, 2009). A subsequent study in humans incorporated this aspect into the experimental design.

## 5 EVIDENCE OF SIMPLE ADDITION OR HYPO‐ADDITION USING REBREATHING METHODS IN HUMANS

Using a rebreathing approach, Guluzade et al. (2023) also assessed respiratory chemoreceptor interaction in awake humans. They elicited progressive CO_2_ ramps at different levels of clamped end‐tidal O_2_ – with hyperoxia to silence the carotid bodies and various levels of hypoxia to activate them. Each rebreathing CO_2_ ramp was preceded by a 5‐min period of hyperventilation such that each CO_2_ ramp began from a hypocapnic range. The HVR was taken as the difference in ventilation between the hyperoxic condition (central chemoreflex only) and three different hypoxic conditions (central + peripheral chemoreflex) at various extrapolated levels of isocapnic $P_{CO_{2}}$. The HVR at each isocapnic step was plotted against the respective $S_{{\mathrm{pO}}_{2}}$, and the slope of the linear relationship was determined as the peripheral chemoreflex sensitivity (PChS) at that $P_{CO_{2}}$. Next, the PChS was calculated at intervals of 1 mmHg over the participant‐specific range of $P_{\mathrm{ETC}O_{2}}$ to produce a PChS versus isocapnic $P_{\mathrm{ETC}O_{2}}$ relationship (Guluzade et al., 2023). In the absence of central chemoreflex input or at fixed central $P_{CO_{2}}$, the peripheral chemoreflex response to low O_2_ increases linearly with higher arterial $P_{CO_{2}}$. But with this method, the relationship quantifies the impact of increasing arterial and progressively heightened central $P_{CO_{2}}$ on the peripheral chemoreflex. Thus, a linear PChS versus $P_{\mathrm{ETC}O_{2}}$ relationship indicates no interaction, whereas a polynomial relationship indicates a progressive effect of central chemoreceptor excitation on the peripheral chemoreflex. Given the high proportion of linear responses, Guluzade et al. (2023) conclude that the interaction between central and peripheral chemoreceptors is additive in ventilation, with variability in the interaction in other parameters (rate and volume), and with hypo‐additive interactions in ventilation only present in female participants. This unique and novel approach to assess chemoreceptor interaction in healthy humans suggests that variability in chemoreflexes is the norm, and may be affected by both ventilatory pattern and biological sex.

## 6 RECONCILIATION AND FUTURE DIRECTIONS

Reduced animal preparations represent invaluable tools to assess the isolated contributions and interactions of afferent inputs on efferent outputs, and they gave the first clues that chemoreflex interactions may not be merely a fixed feature of respiratory control. However, the caveats of these reduced systems, such as the potential effects of species, anaesthesia, decerebration, artificial perfusion, vagotomy and/or carotid body resection, may confound the interpretation of results that differ between research groups using a variety of models and protocols. Thus, creative experimental designs utilized to perturb the system in intact animals and humans, while not without their own challenges, provide an important way forward in understanding afferent integration and respiratory chemoreflex responses. Importantly, as demonstrated by Milloy et al. (2022) and Guluzade et al. (2023), the use of novel in vivo approaches is likely to be particularly well‐suited to map the normative distribution of chemoreceptor interaction across a variety of biological states (e.g., respiratory pattern, sleep–wake state‐dependence, age, and biological sex and/or circulating sex hormones) in humans.

## AUTHOR CONTRIBUTIONS

All authors have read and approved the final version of this manuscript and agree to be accountable for all aspects of the work in ensuring that questions related to the accuracy or integrity of any part of the work are appropriately investigated and resolved. All persons designated as authors qualify for authorship, and all those who qualify for authorship are listed.

### CONFLICT OF INTEREST

The authors declare no conflict of interest.

## References

[eph13819-bib-0001] Blain, G. M. , Smith, C. A. , Henderson, K. S. , & Dempsey, J. A (2010). Peripheral chemoreceptors determine the respiratory sensitivity of central chemoreceptors to CO_2_ . The Journal of Physiology, 588(Pt 13), 2455–2471. 20421288 10.1113/jphysiol.2010.187211 PMC2915520

[eph13819-bib-0002] Day, T. A. , & Wilson, R. J. A (2009). A negative interaction between brainstem and peripheral respiratory chemoreceptors modulates peripheral chemoreflex magnitude. The Journal of Physiology, 587(Pt 4), 883–896. 19103684 10.1113/jphysiol.2008.160689 PMC2669977

[eph13819-bib-0003] Guluzade, N. A. , Huggard, J. D. , Duffin, J. , & Keir, D. A. (2023). A test of the interaction between central and peripheral respiratory chemoreflexes in humans. The Journal of Physiology, 601(20), 4591–4609. 37566804 10.1113/JP284772

[eph13819-bib-0004] Milloy, K. M. , White, M. G. , Chicilo, J. O. C. , Cummings, K. J. , Pfoh, J. R. , & Day, T. A. (2022). Assessing central and peripheral respiratory chemoreceptor interaction in humans. Experimental Physiology, 107(9), 1081–1093. 35766127 10.1113/EP089983

[eph13819-bib-0005] Smith, C. A. , Blain, G. M. , Henderson, K. S. , & Dempsey, J. A (2015). Peripheral chemoreceptors determine the respiratory sensitivity of central chemoreceptors to CO_2_: Role of carotid body CO_2_ . The Journal of Physiology, 593(18), 4225–4243. 26171601 10.1113/JP270114 PMC4594294

